# Development of pro-apoptotic peptides as potential therapy for peritoneal endometriosis

**DOI:** 10.1038/ncomms5478

**Published:** 2014-07-22

**Authors:** K. Sugihara, Y. Kobayashi, A. Suzuki, N. Tamura, K. Motamedchaboki, C.-T. Huang, T.O. Akama, J. Pecotte, P. Frost, C. Bauer, J.B. Jimenez, J. Nakayama, D. Aoki, M.N. Fukuda

**Affiliations:** 1 Department of Obstetrics and Gynecology, Hamamatsu University School of Medicine, 1-20-1 Handayama, Higashi-ku, Hamamatsu, Shizuoka 431-3192, Japan; 2 Tumor Microenvironment, Cancer Center, Sanford-Burnham Medical Research Institute, 10901 North Torrey Pines Road, La Jolla, California 92037, USA; 3 Department of Obstetrics and Gynecology, Keio University School of Medicine, Shinanomachi 35, Tokyo 160-8582, Japan; 4 Southwest National Primate Research Center, Texas Biomedical Research Institute, 7620 NW Loop 410, San Antonio, Texas 78245, USA; 5 Department of Molecular Pathology, Shinshu University Graduate School of Medicine, Asahi 3-1-1, Matsumoto 390-8621, Japan

## Abstract

Endometriosis is a common gynaecological disease associated with pelvic pain and infertility. Current treatments include oral contraceptives combined with nonsteroidal anti-inflammatory drugs or surgery to remove lesions, all of which provide a temporary but not complete cure. Here we identify an endometriosis-targeting peptide that is internalized by cells, designated z13, using phage display. As most endometriosis occurs on organ surfaces facing the peritoneum, we subtracted a phage display library with female mouse peritoneum tissue and selected phage clones by binding to human endometrial epithelial cells. Proteomics analysis revealed the z13 receptor as the cyclic nucleotide-gated channel β3, a sorting pathway protein. We then linked z13 with an apoptosis-inducing peptide and with an endosome-escaping peptide. When these peptides were co-administered into the peritoneum of baboons with endometriosis, cells in lesions selectively underwent apoptosis with no effect on neighbouring organs. Thus, this study presents a strategy that could be useful to treat peritoneal endometriosis in humans.

## Abstract (web summary)

Endometriosis is a painful condition in which endometrial cells are found outside the womb. Here, the authors identify a peptide that specifically binds to a receptor expressed on endometrial epithelial cells and use it to induce apoptosis in both cultured cells and baboons with endometriosis.

## Graphical abstract

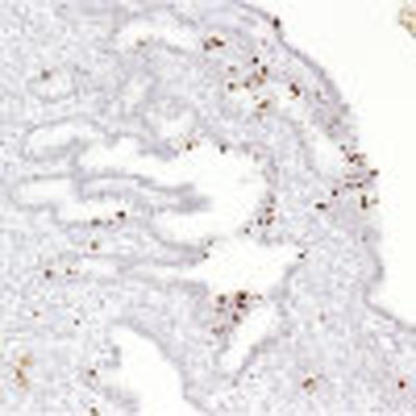

Endometriosis is a commonly encountered gynaecologic disease requiring medical or surgical therapy. As many as 6–10% of women of reproductive age are considered affected by this disease^1^,^2^. Although endometriosis can appear benign and its symptoms usually disappear in post-menopausal women, resultant infertility and severe, disabling pain significantly diminish patients’ quality of life^3^,^4^.

Endometriosis occurs only in higher primates, including humans and baboons. Spontaneous endometriosis reportedly occurs in about 25% of captive baboons, and its prevalence increases with captivity duration^5^. Experiments in non-human primates show a clear positive correlation between endometriosis and a diet containing the chemical dioxin^6^,^7^, which may promote endometriosis by acting as an estrogen-like factor. In patients with endometriosis, many of the pathologic processes including inflammation, the immune response, angiogenesis and apoptosis are all favoured for promoting endometriosis in a manner dependent on steroid hormones^8^,^9^. Thus, in the past, most treatment was via therapeutics-targeting steroid hormones and their receptors^10^,^11^. These drugs can only be administered for a short term due to side effects. Current first-line therapy is oral contraceptive pills, which halt an ovulation and suppress endometriosis tissue growth with minimum side effects^12^. Oral contraceptive pills are also administered with nonsteroidal anti-inflammatory drugs, further reducing endometriosis-associated pain^13^,^14^. Nonetheless, these treatments do not remove endometriosis, and patients with severe symptoms must undergo surgery. However, even then symptoms can recur, requiring multiple surgeries in many cases^15^. Thus, it is critical to develop new strategies to cure this disease, particularly to prevent recurrence after surgery.

One proposed cause for endometriosis is through retrograde menstrual reflux of the endometrium that becomes implanted in regions of the pelvis^16^, most commonly on the ovaries and areas facing the peritoneum^16^,^17^. Thus, we hypothesized that a molecule specifically expressed on the endometrial surface could be targeted by a peritoneally injected drug if that marker is not expressed by other peritoneal surfaces. We report here the identification of such a reagent, namely, a 9-mer peptide that specifically binds to glandular epithelial cells of endometriosis in peritoneum. We also identified its receptor as the cyclic nucleotide-gated channel β3 (CNGB3) and confirmed its expression in endometriosis. On the basis of these findings, we developed potential therapeutics for endometriosis-targeting disease lesions at peritoneal surfaces and tested their activity in baboon endometriosis models. This study presents a strategy that could be useful to treat peritoneal endometriosis in humans.

## Results

### Identification of peptides targeting peritoneal endometriosis

**Figure 1:**
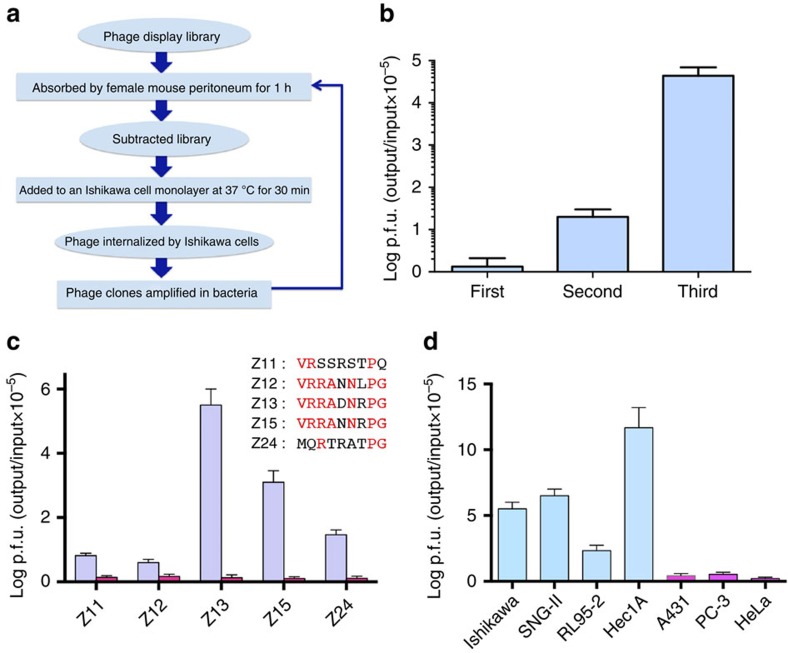
Identification of an endometriosis-targeting peptide by subtractive phage library screening. (**a**) Illustration of library screening steps. A peptide displaying phage library was selected by subtraction using female mouse peritoneum followed by adhesion to and internalization by Ishikawa cells. (**b**) Binding efficiency of phage pools obtained after each screening round assessed by plaque-forming assay. (**c**) Binding of cloned phage to Ishikawa (purple bars) and control A431 (red bars) cells. (**d**) Z13 phage binding to endometrial cell lines (blue bars) and control non-endometrial cell lines (pink bars). In **c**,**d**, each cloned phage was added to a monolayer of each cell line at 37 °C for 30 min. Internalized phage was counted by a plaque-forming assay. Experiments shown in panels **b**–**d** were repeated three times. Each error bar represents s.d.

As yet, there is neither an *in vitro* model nor an *in vivo* animal model available to study endometriosis, other than non-human primates. To identify a peptide that specifically binds to epithelial cells in endometriosis, we hypothesized that some, if not all, human endometrial adenocarcinoma cell lines would express cell surface proteins expressed in glandular epithelial cells in endometriosis. To devise a probe that specifically binds to the endometriosis surface but not to the surface of other organs facing the peritoneum, we undertook subtractive phage library screening^18^,^19^. A T7 phage-based library (10^9^ clones, 10^11^ plaque-forming unit) of linear 9-mer peptides was injected into the peritoneal cavity of a female mouse to allow absorption of phage clones to the peritoneal surface *in vivo* for 1 h. The precleared library was recovered from peritoneal fluid and added to a monolayer of human endometrial adenocarcinoma Ishikawa cells cultured *in vitro*. We choose Ishikawa cells as this cell line shares characteristics with mature endometrial epithelial cells^20^,^21^. We also wanted to identify a peptide internalized by endometrial glandular epithelial cells so that a drug conjugated with that peptide would penetrate target cells. We therefore incubated phage with live Ishikawa cells at 37 °C for 30 min to facilitate phage internalization. Selected phage clones were recovered after solubilizing cells with detergent and amplified in bacteria (Fig. 1a). After three rounds of subtractive library screening, the number of phage clones with Ishikawa cell-binding activity relative to the total number of added phage increased 10,000-fold (Fig. 1b).

The third screen-positive phage pool was overlayed on frozen tissue sections of endometriosis lesions surgically isolated from endometriosis patients. Immunohistochemistry using an anti-phage antibody showed positive signals on glandular epithelial cells, particularly at apical cell surfaces in endometriosis (Supplementary Fig. 1). These results suggest that, despite differences between endometriosis and endometrial adenocarcinoma, the positive phage pool bound to the Ishikawa cancer cell line contained a clone or clones binding to endometrial glandular epithelial cells.

Sequencing of insert DNAs from isolated phage clones revealed the deduced consensus sequence VRRAXNXPG (where X represents a varying amino-acid residue; Fig. 1c). The presence of the consensus sequence attests to the high specificity of selected clones. *In vitro* binding assays indicated that each clone bound to Ishikawa cells at higher efficiency than to control skin epidermoid carcinoma A431 cells. Among the clones, z13, which displayed the sequence VRRADNRPG, was the strongest binder. Z13 phage bound to endometrial adenocarcinoma SNG-II, RL95-2 and Hec1A cells but not to 431, prostate cancer PC3 or cervical cancer HeLa cells (Fig. 1d).

**Figure 2:**
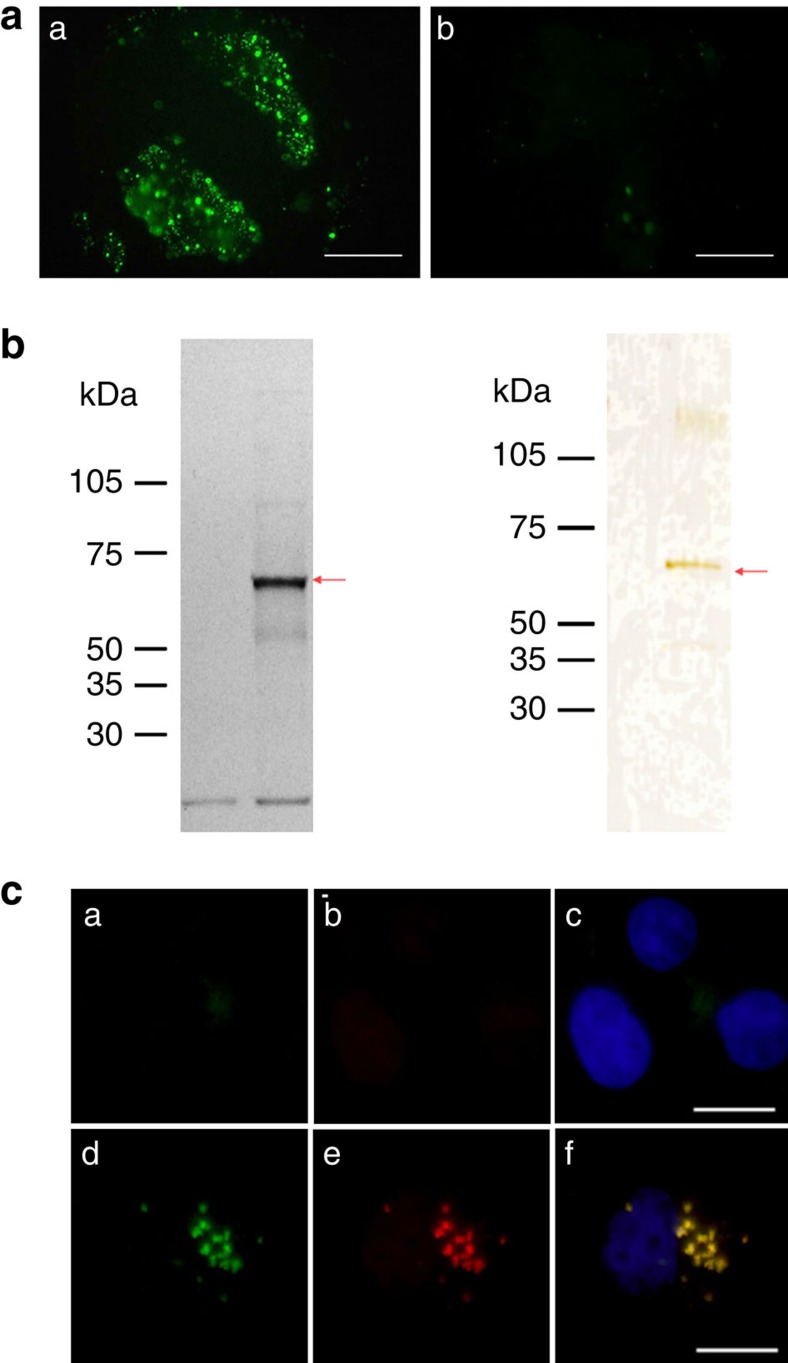
Identification of z13 peptide receptor. (**a**) Fluorescence micrographs of Ishikawa cells (left) and control A431 cells (right) overlayed with a synthetic z13 peptide tagged with fluorescein isothiocyanate (FITC) and left at 37 °C for 15 min. Scale bar, 50 μm. (**b**) Visualization and isolation of the z13 peptide receptor. Left: cell surface proteins expressed on Ishikawa cells were biotinylated. Cell lysates were bound to z13 peptide-conjugated agarose beads, and bound proteins were eluted with irrelevant peptide (lane 1) or z13 peptide (lane 2). Biotinylated proteins in each eluate were detected by peroxidase-conjugated avidin and a luminescent peroxidase substrate. Right: silver staining of peptide-affinity-purified z13 receptor from endometriosis. Endometriosis tissues isolated from patients were homogenized, and microsome membrane fraction was prepared. Proteins solubilized with detergent were applied to a z13 peptide-conjugated agarose column, and bound proteins were eluted with irrelevant peptide (lane 1) or z13 peptide (lane 2). Proteins in each eluate in SDS–PAGE were detected by silver staining. (**c**) Fluorescence micrographs of HeLa cells transfected with control empty vector (upper row) or with an expression vector encoding CNGB3–MYC (lower row). Binding of FITC-z13 peptide (green) to HeLa cells transfected with mammalian expression vectors (a,d), immunostained with anti-MYC followed by Alexa 549 (red)-conjugated anti-mouse IgG antibody (b,e) and merged images including 4',6-diamidino-2-phenylindole (blue) to indicate nuclear staining (c,f). Scale bar, 20 μm.

To confirm z13 peptide-binding activity, we chemically synthesized z13 peptide with an amino-terminal fluorescein isothiocyanate (FITC) tag and added to Ishikawa cells and control A431 cells (Fig. 2a). Fluorescence micrographs showed binding of FITC-z13 to Ishikawa cells, but not to A431 cells. Micrograph of Ishikawa cells showed a punctate cytoplasmic staining pattern, suggesting that z13 is internalized to endosomes.

### Identification of the z13 peptide receptor

To develop a clinically relevant therapeutic strategy, we searched for the z13 peptide receptor. To do so, Ishikawa cells were surface biotinylated and lysed, and then lysates were incubated with z13 peptide-conjugated agarose beads. Bead-bound materials were then eluted by z13 peptide, and biotinylated proteins detected by avidin blot (Fig. 2b, left), revealing a single 68-kDa protein. To identify this protein, the microsomal membrane fraction was prepared from endometriosis tissue surgically removed from patients. Membrane proteins solubilized with detergent were applied to a z13 peptide-affinity column and column-bound materials were eluted by z13 peptide. A silver-stained gel revealed a 68-kDa protein (Fig. 2b, right), and proteomic analysis identified the peptide sequence, QRTALYK (Supplementary Figs 2 and 3; Supplementary Table 1), which is unique to the CNGB3 (Supplementary Fig. 4). CNGB3 protein has six transmembrane domains^22^. A large part of this protein is buried in the lipid bilayer, the N- and C-terminal domains are cytoplasmic and presumably the z13-binding regions are extracellular.

To assess binding of FITC-z13 to CNGB3, we transfected HeLa cells with a mammalian expression vector encoding a CNGB3–MYC fusion protein or with control empty vector. FITC-z13 did not bind to vector-transfected control cells (Fig. 2c(a–c)). HeLa cells transfected with the CNGB3–MYC expression construct and stained by anti-MYC antibody showed an endosome-like pattern (Fig. 2c(e)). When FITC-z13 was added to culture medium of CNGB3–MYC-expressing HeLa cells, FITC-z13 bound to sites marked by MYC epitope expression (Fig. 2c(d–f)). The kinetics of FITC-z13 binding to CNGB3–MYC-expressing HEK293T cells showed a *K*_d_ of 9.759 × 10^−6^ M and a *B*_max_ of 0.9086 moles per receptor (Supplementary Fig. 5). These results indicate that recombinant CNGB3 expressed in a mammalian cell has z13 peptide-binding activity on the cell surface and internalized to endosome, supporting the hypothesis that CNGB3 is the z13 receptor.

**Figure 3:**
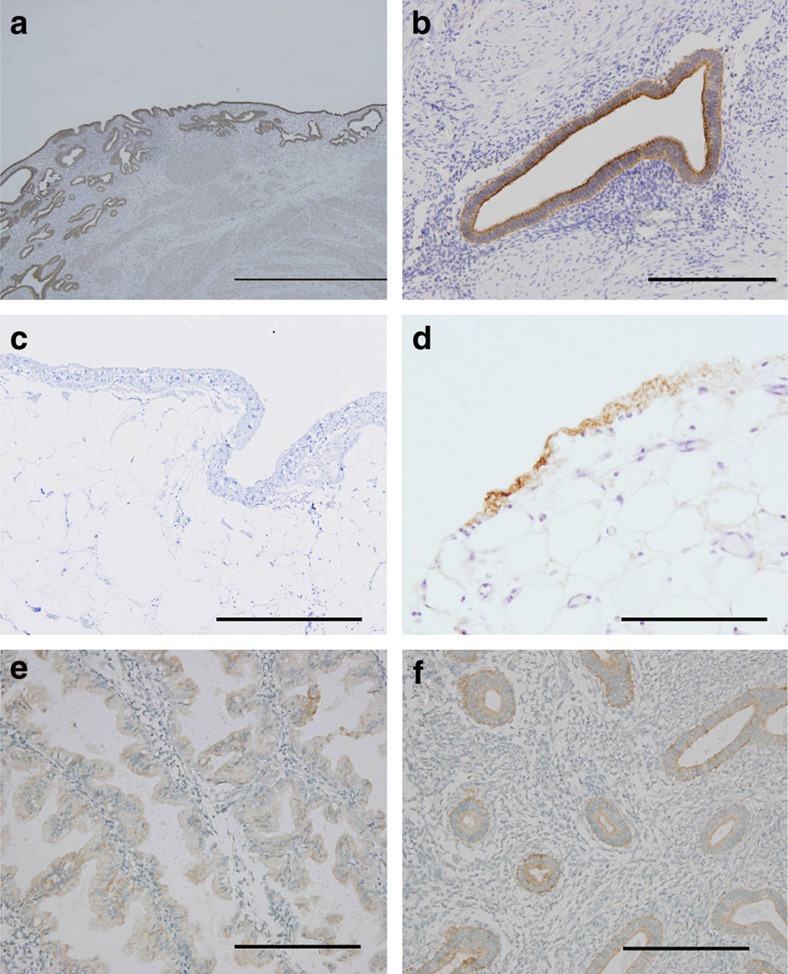
Immunohistochemistry of paraffin tissue sections using mouse monoclonal anti-CNGB3 antibody. (**a**) Endometriosis lesions from an endometriosis patient. Scale bar, 500 μm. (**b**) An endometrial gland in endometriosis tissue. Scale bar, 200 μm. (**c**) Peritoneal tissue from a cycling woman without endometriosis. Scale bar, 100 μm. (**d**) Peritoneal tissue from an endometriosis patient. Scale bar, 100 μm. (**e**) Uterine endometrium at secretory phase. Scale bar, 200 μm. (**f**) Uterine endometrium at proliferative phase. Scale bar, 200 μm.

To examine CNGB3 protein expression in endometriosis, we generated a mouse monoclonal antibody against a peptide sequence of human CNGB3, corresponding to K^721^ to P^750^ within the cytoplasmic domain (Supplementary Fig. 4). Antibody specificity was validated by immunostaining of CNGB3–MYC expressed in HeLa cells (Supplementary Fig. 6). This antibody robustly stained glandular epithelia of endometriosis in tissue sections (Fig. 3a,b). When we evaluated endometriosis tissue sections from 35 endometriosis patients, we found that 31 specimens showed strongly positive immunostaining and 4 showed weak/negative immunostaining. Peritoneal surfaces from cycling women without endometriosis were not stained by this antibody, whereas those from endometriosis patients were stained by this antibody (Fig. 3c,d), suggesting that endometrial cells are spread across a wide area on the peritoneum of endometriosis patients. Eutopic endometrial tissues at secretory and proliferative phases were weakly stained by this antibody (Fig. 3e,f). Immunohistochemistry of human tissues showed that this antibody did not stain the surface of organs facing the peritoneal cavity (data not shown).

### Induced apoptosis of CNGB3-expressing cells

**Figure 4:**
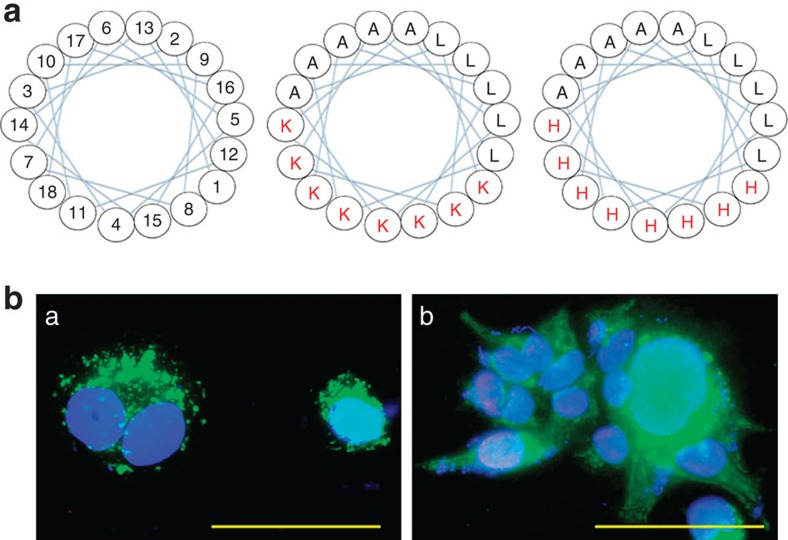
Design and activity of endosome-escaping peptide. (**a**) Helical wheels of alpha helix (left), and alignment of KLAKLAKKLAKLAKKLAK (center) and HLAHLAHHLAHLAHHLAH (right). Note that polar or hydrophilic amino acids are concentrated on one side of helix and hydrophobic amino acids on the other. (**b**) Endosome-escaping activity of HLAH-z13. Biotin-z13 was bound to CNGB3–MYC-expressing HEK293T cells at 37 °C for 30 min with (b) or without (a) HLAH-z13. Cells were stained by Alexa 488-conjugated avidin (green) followed by 4',6-diamidino-2-phenylindole (blue) nuclear staining. Scale bar, 50 μm.

The above results suggest that z13 peptide can be used to deliver a drug against endometriosis expressing CNGB3. For a potential therapeutic, we chose the pro-apoptotic 18-mer peptide, KLAKLAKKLAKLAKKLAK, abbreviated KLAK in this study, which was devised from [KLAKLAK]_2_ peptide^23^,^24^,^25^. In these types of peptides, one side of alpha helix is hydrophilic and the other side is hydrophobic (Fig. 4a, centre), which has the overall effect of disrupting mitochondrial membranes. In dKLAK-z13, the KLAK moiety was made using d-amino acids to prevent proteolysis, as described by Ellerby *et al.*^24^

When dKLAK-z13 was added to culture medium of HEK293T cells transfected with expression vector for CNGB3–MYC, however, apoptosis was not induced. We hypothesized that this lack of effect could be due to the fact that receptor-bound dKLAK-z13 localizes to the luminal side of endosomes, a prediction supported by the endosome-like pattern of fluorescent z13 (Fig. 2c(d–f)). To release dKLAK-z13 from the endosomes to the cytoplasm, we devised an endosome-escaping 18-mer peptide HLAHLAHHLAHLAHHLAH (here abbreviated as HLAH) by replacing lysines in KLAKLAK with histidines (Fig. 4a, right) to provide histidine-rich sequences capable of destabilizing endosome membranes at acidic pH^26^,^27^. When biotinylated z13 was bound to HEK293T cells overexpressing CNGB3–MYC protein, biotin-z13 showed a punctate endosome/lysosome-like pattern (Fig. 4b(a)), whereas it showed diffuse cytoplasmic signals in the presence of HLAH-z13 (Fig. 4b(b)), suggesting that HLAH-z13 promotes endosome-escaping activity releasing biotin-z13 from endosomes to the cytoplasm. In CNGB3-expressing HEK293T cells, lysosomal marker acid phosphatase localization in lysosomes was lost in cells cultured in a medium containing HLA-z13 (Supplementary Fig. 7).

**Figure 5:**
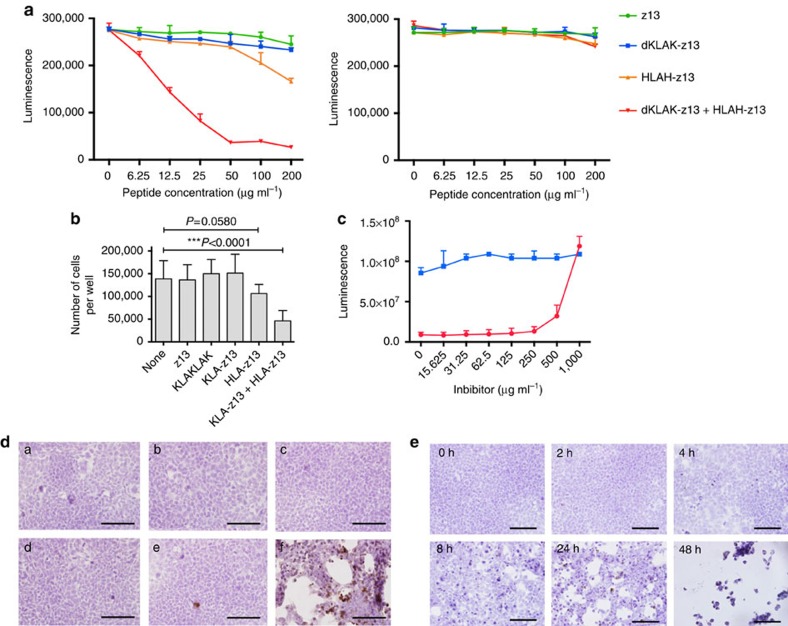
Z13-targeted apoptosis of CNGB3-expressing cells *in vitro*. (**a**) Cell viability assays of CNGB3-positive A431 cells (left) and control CNGB3-negative A431 cells (right) after incubating cells with z13, dKLAK-z13, HLAH-z13 or dKLAK-z13 plus HLAH-z13. Cells were cultured in a medium containing each peptide at the indicated concentrations at 37 °C for 6 h. ATP levels were measured by CellTiter Glo (Promega). dimethylsulphoxide alone at 37 °C for 6 h at a final concentration of 0.01% had no effect by this assay (data not shown). (**b**) Number of living CNGB3-positive and -negative A431 cells following treatment with the indicated reagents for 20 h. (**c**) Inhibition of the activity by KLA-z13 plus HLA-z13 by palmitoyl-z13 peptide. CNGB3-expressing A431 cells were treated with C16-z13 alone (blue) or with a mixture of KLAK-z13 (100 μg ml^−1^) and HLA-z13 (25 μg ml^−1^; red) at 37 °C for 6 h. Each data point represents average of triplicate measurements. This analysis was repeated three times. (**d**) TUNEL assay of CNGB3–MYC-expressing HEK293T cells cultured in a medium containing z13 peptide with or without an apoptosis-inducing peptide and/or endosome-escaping peptide. Cells were cultured at 37 °C for 20 h in a medium containing: no peptide (a); z13, 100 μg ml^−1^ (b); dKLAK, 100 μg ml^−1^ (c); dKLAK-z13, 100 μg ml^−1^ (d); HLAH-z13, 25 μg ml^−1^ (e); and dKLAK-z13, 100 μg ml^−1^, plus HLAH-z13, 25 μg ml^−1^ (f). Scale bar, 100 μm. (**e**) TUNEL assay of CNGB3–MYC-transfected HEK293T cells treated with a mixture of dKLAK-z13 (100 μg ml^−1^) and HLAH-z13 (25 μg ml^−1^) for the periods indicated. Scale bar, 100 μm.

We next assessed cytotoxic activity of dKLAK-z13 in the presence of HLAH-z13 using a cell viability assay (Fig. 5a,b). When CNGB3–MYC-transfected A431 cells were cultured in a medium containing z13, dKLAK-z13 or HLAH-z13 at 37 °C for 6 h, cell viability was not affected, except at high concentrations of dKLAK-z13 (>200 μg ml^−1^) or HLAH-z13 (>50 μg ml^−1^). By contrast, a mixture of dKLAK-z13 and HLAH-z13 each at a low concentration showed clear cytotoxicity, but had no toxic effect on control CNGB3-negative A431 cells. Cytotoxicity of KLAK-z13 plus HLA-z13 was blocked by excess z13 (Fig. 5c).

When CNGB3-expressing HEK293T cells were cultured in a medium containing z13 (100 μg ml^−1^), dKLAK-z13 (100 μg ml^−1^) or HLAH-z13 (25 μg ml^−1^) at 37 °C for 20 h, cells did not undergo apoptosis (Fig. 5d(a–e)). By contrast, a mixture of dKLAK-z13 (100 μg ml^−1^) and HLAH-z13 (25 μg ml^−1^) promoted massive apoptosis and cell detachment (Fig. 5d(f)). The same mixture showed time-dependent cytotoxicity in CNGB3-expressing HEK293T cells (Fig. 5e), whereas it had no effect on control A431 cells (data not shown). These results suggest that a mixture of dKLAK-z13 and HLAH-z13 specifically targets CNGB3-expressing cells to induce apoptotic cell death.

### Targeted apoptosis of peritoneal endometriosis *in vivo* in the baboon

We tested the effect of a mixture of dKLAK-z13 and HLAH-z13 peptides on baboon endometriosis models *in vivo*, as baboon endometriosis tissues were stained by anti-CNGB3 antibody in a similar manner as human endometriosis lesions (Fig. 3a,b) are stained (Supplementary Fig. 8). Six female baboons with advanced endometriosis were identified based on clinical symptoms, which include infertility and behaviour consistent with the inability to conceive, namely, dysmenorrhoea and extended menses. Under general anaesthesia, a 5-mm diagnostic laparoscope was used to inspect pelvic organs, which confirmed endometriosis (Supplementary Fig. 9). After washing the peritoneal cavity with phosphate-buffered saline (PBS), a mixture of dKLAK-z13 (100 μg ml^−1^) and HLAH-z13 (25 μg ml^−1^) peptides dissolved in PBS was injected into the peritoneum of three baboons. Animals were left ~20 h after administration of the peptides to allow for apoptosis to occur. Endometriosis lesions and surrounding peritoneal tissues were collected post mortem. Endometriosis tissue collected from three baboons without drug injection were used as control.

**Figure 6:**
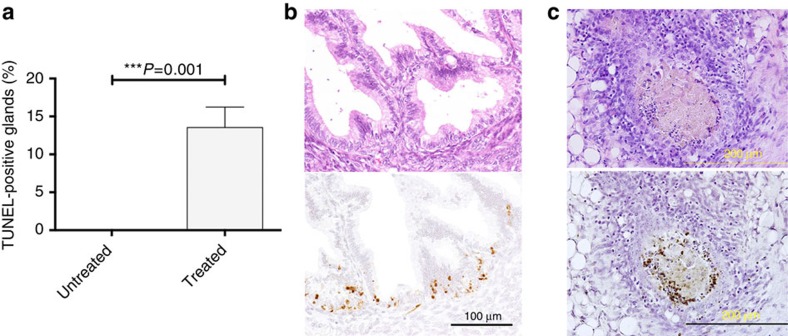
Induction of apoptosis in baboon endometriosis tissue *in vivo*. (**a**) Quantitative TUNEL analysis. Shown is the percentage of TUNEL-positive endometrial glands in tissues from three control untreated baboons compared with three animals treated with a mixture of dKLAK-z13 and HLAH-z13 peptides (see Methods). The number of glands counted and the number of TUNEL-positive glands (indicated in parentheses) were 5 (0), 8 (0) and 18 (0) for untreated and 8 (1), 18 (2) and 26 (3) for treated. Percentages of TUNEL-positive glands from untreated and treated animals were 0.00%±0.00 (*n*=3) and 13.53%±1.56 (*n*=3), respectively. (**b**) TUNEL assay of baboon endometriosis. Upper panels: hematoxylin and eosin-stained images. Lower panels: TUNEL assay of corresponding areas. Left: an endometrial gland in the ovary. Scale bar, 100 μm. Right: TUNEL-positive cell debris in an endometrial gland in the omentum. Scale bar, 200 μm.

Histology of hematoxylin and eosin-stained tissue sections revealed evidence of endometriosis in all three animals treated with a mixture of dKLAK-z13 and HLAH-z13 peptides. Terminal deoxynucleotidyl transferase-mediated dUTP nick end labeling (TUNEL) assays of endometriosis lesions collected from three untreated control baboons revealed no TUNEL positivity in gland tissue (Fig. 6a, left column). By contrast, we found TUNEL-positive glands in tissues collected from all three baboons treated with the dKLAK-z13 and HLAH-z13 peptide mixture (Fig. 6a, right column). Those signals were seen in glandular epithelial cells in ovarian endometriosis and in the lumen of endometrial gland in the omentum (Fig. 6b). No evidence of apoptosis was detected in cells facing peritoneum in liver, kidney, spleen, colon and stomach (data not shown). These results show overall, as proof of concept, that z13-targeted induced apoptosis occurred in endometriosis model in baboons *in vivo.*

## Discussion

A general strategy of translational research is to identify a gene product associated with a disease of interest and then develop drug targeting that molecule. However, in identifying peptides that target endometriosis but not non-endometrial cells facing the peritoneal cavity, we took a novel approach that does not require knowledge of a target. We also demonstrated that CNGB3 is the receptor for z13 peptide based on its expression pattern, not its function. We feel that analysing CNGB3 function in detail is not a priority for clinical application of this work.

Endometriosis is notoriously resistant to apoptosis^28^,^29^. By combining endometriosis-targeting peptide with pro-apoptotic KLAKLAK peptide^23^,^24^ and that with newly developed potent endosome-escaping HLAHLAH peptide (Fig. 4), we were able to induce apoptosis in cells of endometriosis *in vivo* in baboon models. However, we acknowledge that a potentially informative control is missing from this experiment. As we used untreated baboons as controls for this experiment and did not administer a randomized control peptide, it currently remains unclear how much of the induction of apoptosis is attributable specifically to the use of the z13 peptide. Use of two z13 chimeric peptides means that the peptides will compete for the receptor, which may not be the ideal therapeutic strategy. Modifications of the approach, such as use of a KLA-HLA hybrid-type peptide, should be tested in the future. Nonetheless, this study presents proof-of-concept evidence that these reagents can be developed as potential therapeutics.

Phage display technology allows unbiased identification of a factor in the absence of biochemical or cell biological information. The strength of this technology is the very large size of the library, which contains as many as 10^9^ clones. In addition, identified short peptide sequences can be synthesized chemically and modified for further functionality. This technology has been successfully used to identify peptide ligands specific for organ vasculature^30^, tumour vasculature^31^,^32^ and cell adhesion molecules^33^,^34^. In this study, we employed subtractive library screening to identify a peptide z13 that targets endometriosis tissue by eliminating phage clones with binding activity to the peritoneal surface and organs facing the peritoneum (Fig. 1).

The z13 receptor CNGB3 is highly expressed in endometrial glandular epithelial cells and peritoneal surfaces in specimens collected from endometriosis patients (Fig. 3a,b), whereas CNGB3 protein was not detected in peritoneal surfaces (Fig. 3c) and organs facing peritoneum in healthy women. This suggests that peritoneally injected drug with CNGB3-binding activity can target macroscopically invisible endometrial epithelial cells adhering to the peritoneal surfaces (Fig. 3d). Such targeting activity by z13 is significant, as these cells are difficult to be removed by surgery and if they are left in peritoneum they could potentially develop into endometriosis lesions.

When we conjugated z13 peptide with an apoptosis-inducing dKLAK peptide, this construct did not induce apoptosis of CNGB3-expressing cells (Fig. 5a, left, blue line; Fig. 5d(d)). We speculated that dKLAK-z13 binds to CNGB3 on the endometrial glandular cell surface and is internalized to endosomes (Fig. 2a(a); Fig. 2c(d–f); Fig. 4b(a)), but not released to the cytoplasm where mitochondria reside. To circumvent this problem, we developed an endosome-escaping peptide comprised of histidine-rich sequences (Fig. 4a, Fig. 4b(b))^26^,^35^,^36^. The p*K*_a_ of the histidine imidazole group is 6.5, and thus histidine behaves as hydrophobic residue at neutral pH, whereas it is protonated and behaves as a basic peptide in the slightly acidic endosomal milieu. Once protonated, the peptide disrupts endosomal membranes, destabilizing endosomes. We devised an endosome-escaping HLAH peptide by replacing K in KLAK peptide with H (Fig. 4a, right). HLAH peptide devised here could have wide applications to enhance activity of drugs that are not functional when trapped in endosomes. It needs to be emphasized that this strategy should be applicable to any cell surface protein of sorting pathway, regardless of physiological activity of targeting proteins.

Human endometriosis tissues reportedly exhibit minimal apoptosis^29^,^37^. Indeed, apoptosis was not detected in endometriosis tissues in untreated baboons (Fig. 6a, left column). By contrast, in baboons treated overnight with a mixture of dKLAK-z13 and HLAH-z13, significant apoptosis was detected in endometriosis lesions (Fig. 6b,c). However, the effect of z13 was modest (Fig. 6a, right column) *in vivo* compared with its robust activity in cultured cells (Fig. 5). Some loss of activity may be attributable to the peritoneal environment. For example, extracellular matrix may cover the surface of peritoneal tissues, including endometriosis and block access of the peptide to cells. Drugs administered to the peritoneum may also be diluted to sub-optimal doses. If that were the case, an appropriate controlled-release formulation would be required to maintain optimal z13 concentrations in the peritoneum. Therefore, in the future we will be exploring these and other possibilities to develop clinically relevant approaches to improve z13 efficacy in the peritoneal environment.

While endometriosis is characterized by the presence of both endometrial epithelial and stromal cells, occasionally there is an absence of epithelial cells, as was found in 44.9% of 274 peritoneal biopsies analysed by Boyle *et al.*^38^ In our study, we developed a vehicle to target endometrial epithelial cells. However, development of a similar vehicle that also targets endometrial stromal cells could represent a more efficacious strategy, possibly by targeting the entire lesion. Nonetheless, our findings suggest a novel approach to the treatment of endometriosis, one that awaits further tests of clinical relevance.

## Methods

### Synthetic peptides

The following peptides were synthesized by GenScript (Piscataway, NJ): z13, VRRADNRPG; irrelevant peptide, RGMSDTTAL; fluorescent FITC-z13; dKLAKLAK-z13, *KLAKLAKKLAKLAKKLAK*VRRADNRPG (d-amino acids italicized); HLAH-z13, HLAHLAHHLAHLAHHLAHVRRADNRPG; and CNGB3 antigen, KENEDKGKENEDKDKGREPEEKP (3B2 epitope).

### Human subjects

Endometriosis specimens including peritonial tissues were obtained from patients undergoing hysterectomy for uterine myomas and ovarian cysts after each patient provided written informed consent. The use of human subjects in this study was approved by the Institutional Review Board of Keio University School of Medicine. Formalin-fixed and paraffin-embedded tissue blocks of normal endometrium with adenomyosis (three patients) and peritoneum without endometriosis (four patients) were retrieved from a pathology file of Department of Laboratory Medicine, Shinshu University Hospital. The Institutional Review Board of Shinshu University School of Medicine approved the study plans, and granted a waiver of informed consent, since diagnostic use of the samples was completed before this study and there was no risk to the patients. All samples were number coded to protect patient confidentiality.

### Use of mice

The protocols for monoclonal antibody production and use of mice for phage library screening were approved by the Institutional Animal Care and Use Committee (IACUC) of the Sanford-Burnham Medical Research Institute.

### Cell lines and culture

All cells were cultured in DME/F12 (50:50) mixed medium supplemented with 10% fetal calf serum, 2 mM glutamine, 1 mM pyruvate, 100 U ml^−1^ penicillin and 100 μg ml^−1^ streptomycin at 37 °C in a humidified incubator under 5% CO_2_.

### Phage library screening

A T7 phage library displaying random 9-mer peptide sequences constructed in the T7 Select 415-1b vector (Novagen, Madison, WI) was provided by Dr E. Ruoslahati, Sanford-Burnham Medical Research Institute. A 6-week-old C57/BL6 female mouse was anaesthetized with avertin, and 1 ml of the T7 phage library containing a total of 10^11^ clones was injected into the peritoneal cavity and left for 1 h. The library was recovered by washing the peritoneal cavity with 10 ml of 20 mM sodium phosphate buffer, pH 7.4, containing 0.15 M sodium chloride (PBS). This subtracted library was added to a monolayer of Ishikawa cells grown in a 3.5-cm tissue culture plate and incubated at 37 °C for 30 min, allowing bound phage to be internalized by endocytosis. Cells were washed six times with DMEM and detached by trypsinization. Cells were solubilized by 1% NP-40 in PBS, and competent BL21 bacteria were added. Phage-infected bacteria were cultured at 37 °C until lysis occurred. The amplified phage, 1 × 10^7^ clones (1 ml), was subtracted again using mouse peritoneum and selected by incubation with Ishikawa cells as described. This cycle was repeated three times. Binding of each cloned phage to target cells was determined by counting the number of phage plaques recovered from cells relative to the phage numbers added to cells. Sequencing of the phage clone was performed as described^37^.

### Phage immunohistochemistry using frozen human tissue sections

Tissues were fixed with 20% buffered formalin (pH 7.4) for 48 h and then incubated with a 0.88 M hypertonic gum-sucrose solution overnight. The tissues were immediately frozen in O.C.T. compound (Sakura Finetechnical, Tokyo, Japan) at −80 °C and sliced at 6 μm thickness. Frozen sections were placed on slides and stored frozen until use. Phage was overlaid on the sections at room temperature for 30 min, and sections were then washed with PBS and fixed with 1% paraformaldehyde (PFA) in PBS. Immunohistochemistry was undertaken using a rabbit anti-T7 phage antibody followed by immunoperoxidase reactions. Polyclonal rabbit anti-T7 phage antibodies were generated by Drs P. Laakkonen, J.A. Hoffman, K. Pokka and E. Ruoslahti, Sanford-Burnham Medical Research Institute, and were provided to us for this study. These antibodies do not stain frozen or paraffin sections of normal human and mouse tissues. Staining was visualized by 3, 3′-diaminobenzidine (DAB) (Zymed/Invitrogen, Carlsbad, CA) and hematoxylin was used for counterstaining.

### Fluorescence microscopy

Ishikawa cells and A431 cells were grown on glass coverslips in 3.5 cm tissue culture plates. Ishikawa cells grown on glass coverslips were incubated in a medium containing FITC-z13 peptide (1 μg ml^−1^) at 37 °C for 15 min. After washes with PBS, cells were fixed with 1% PFA in PBS and inspected under under a Zeiss Axioplan fluorescence microscope. A431 cells transfected with CNGB3–MYC/pcDNA3 vector were cultured in a medium containing HLA-z13 peptide at 50 μg ml^−1^ for 1 h. Cells were fixed with 4% PFA in PBS at room temperature for 15 min, permeabilized with 1% saponin and stained by rabbit anti-ACP1 antibody (LifeSpan Bioscience) followed by Alexa 488-conjugated goat anti-rabbit IgG antibody (Invitrogen).

### Cell surface biotinylation and z13 peptide-affinity chromatography

An Ishikawa cell monolayer was washed three times with PBS. The biotinylation reagent sulpho-NHS-LC-biotin (Pierce, Rockford, IL) was dissolved in PBS and added to cells at 0.5 mg ml^−1^. Thirty minutes later, the monolayer was washed three times with 20 mM Tris-HCl buffer, pH 7.4, containing 0.15 M sodium chloride (TBS). Cells were scraped from plates with a rubber policeman and solubilized in TBS containing 50 mM octyl-thio-glucoside and protease inhibitors (Complete mini, Roche, Indianapolis, IN) at 4 °C. The cell lysate obtained after centrifugation was precleared with agarose beads at 4 °C for 2 h. The precleared lysate was then incubated with z13-conjugated agarose beads, which were prepared by conjugating cys-z13 peptide (1 mg) with sulpho-link agarose beads (1 ml, Pierce). Z13 beads were washed with TBS containing 50 mM octyl-thio-glucoside and bead-bound materials were then eluted with TBS/octyl-thio-glucoside buffer containing z13 peptide (1 mg ml^−1^). Biotinylated proteins eluted from the z13 beads were detected by SDS–polyacrylamide gel electrophoresis (PAGE) followed by peroxidase avidin blot and an ECL (Amersham, Piscataway, NJ) chemiluminescence reaction.

### Z13 peptide-affinity chromatography and proteomics

Surgically isolated endometriosis tissue (2 g) was homogenized in 100 ml TBS containing protease inhibitors (Complete mini, Roche). The homogenate was centrifuged at 2,000 *g* for 15 min, and the resulting supernatant was centrifuged at 100,000 *g* for 60 min. The pellet (membrane fraction) was resuspended in TBS containing 50 mM octyl-thio-glucoside and applied to 50 μl z13-conjugated agarose beads as described above. z13 beads were washed three times with 1.5 ml TBS containing 50 mM octyl-thio-glucoside and then washed three times with 50 μl of the same buffer containing irrelevant peptide (1 mg ml^−1^). Bead-bound materials were eluted with 50 μl of buffer containing z13 peptide (1 mg ml^−1^). Eluted proteins were resolved on SDS–PAGE and detected by silver staining. A single band at 68 kDa was excised from the gel, digested with trypsin and analysed by matrix-assisted laser desorption/ionization–time of flight mass spectrometry (MS) at the proteomics facility at the Sanford-Burnham Medical Research Institute. Details of the proteomics procedure and data are provided in Supplementary information (proteomics).

### In-gel digestion and sample preparation for liquid chromatography/MS analysis

A silver-stained SDS–PAGE gel band corresponding to the 68 kDa z13 receptor was cut into 1 mm × 1 mm pieces, transferred to an ethanol-rinsed microcentrifuge tube and destained. Proteins in gel pieces were reduced and alkylated using a final concentration of 5 mM dithithreitol and 15 mM iodoacetamide before digestion with Trypsin (Promega) at 25 ng μl^−1^ in 50 mM ammonium bicarbonate, first for 1 h on ice and then for 16 h at 37 °C using shaking incubator. Peptides were extracted from gels, transferred to a new tube and eluted as follows: 100 μl water was added to gels and samples were sonicated 10 min in a water bath followed by incubation with 5% formic acid in water, four extractions in 50% acetonitrile in 5% formic acid in water, one extraction in 70% acetonitrile and a final extraction in 100% acetonitrile. Extracted peptides were then pooled in an ethanol-rinsed tube, vacuum dried and re-dissolved in 18 μl 0.1% trifluoroacetate. Tryptic peptides were then concentrated and desalted using a Millipore C18 Zip Tip (Millipore). Eluants were then vacuum dried and re-dissolved in 10 μl liquid chromatography (LC)/MS loading buffer (2% acetonitrile in 0.1% formic acid in water).

### Protein identification using one-dimensional LC/MS/MS

Three ml of tryptic-digested samples was loaded onto the automated Nano LC-LTQ MS/MS (Thermo Scientific) using an Eksigent Nano 2D LC system, a switch valve, a C18 trap column (Agilent, Santa Clara, CA) and a capillary reverse-phase column (15 cm Magic C18 AQ resin (Michrom)). An ADVANCE low flow Michrom source (Bruker-Michrom) was used to ionize peptides as they elute from the capillary column in a linear gradient from 100% buffer A (2% acetonitrile in water containing 0.1% formic acid) to 15% buffer A plus 85% buffer B (100% acetonitrile in water containing 0.1% formic acid) over 80 min. Data relevant to the strongest ions above an intensity of 50 × 10e4 were collected with dynamic exclusion enabled and the collision energy set at 35%. Data representing the strongest ions above an intensity of 50 × 10e4 were collected with dynamic exclusion enabled and the collision energy set at 35%.

### Protein identification and data analysis

MS/MS spectra were submitted to Sorcerer 2 (Sage-N Research) analysis using the SEQUEST algorithm as the search programme for peptide/protein identification. SEQUEST was set up to search the target-decoy ipi.Human.v3.16 database containing protein sequences using trypsin as an enzyme, allowing up to two missed cleavages, full tryptic search and precursor mass tolerance of 1.5 amu. Fixed modification was set for cysteines to account for carboxyamidomethylation in case of cysteine alkylation. A differential search included 16 Da for methionine oxidation. Search results were viewed, sorted, filtered and statistically analysed using the proteomics data analysis software Peptide/Protein prophet (ISB).

### Monoclonal anti-CNGB3 antibody and immunohistochemistry

A peptide sequence of human CNGB3, KENEDKGKENEDKDKGREPEEKP-C, was synthesized by GenScript. A cysteine was added at the carboxy terminus for conjugation with keyhole limpet hemocyanin (Immunopure KLH, Pierce) using a SMPB cross-linker (Pierce) according to the manufacturer’s protocol. Female Balb/c mice were immunized with this conjugate, and spleens were isolated from the immunized mice. Lymphocytes released from the spleen were fused to mouse myeloma P3X cells, and hybridoma clones were screened by immunostaining as Ishikawa (positive) and A431 (negative) cells. A hybridoma clone was subcloned by a limited dilution, establishing the 3B2 clone. Paraffin tissue sections endometriosis patients (*n*=35) were obtained from Folio Biosciences (Columbus, OH). A normal uterine endometrium tissue microarray (60 cores) and a normal tissue microarray (60 cores) were obtained from Imgenex (San Diego, CA). Antigen retrieval was performed by autoclaving sections at 110 °C for 1 min in antigen retrieval solution (Vector Laboratories). Immunohistochemistry with 3B2 (mouse IgG) was performed using an ABC kit (Vector Laboratories) with a DAB colour reaction and hematoxylin counterstaining.

### Expression of recombinant CNGB3–MYC protein

CNGB3 complementary DNA^39^ was kindly provided by Dr Hisao Ueyama, Department of Ophthalomology, Shiga University of Medical Science, Japan. cDNA encoding full-length CNGB3 was excised by HindIII and BsrGI and ligated into the HindIII and Asp718I sites of pcDNA3.1/myc-His-B vector (Invitrogen). HeLa cells were transfected with this vector, and expression of CNGB3–MYC/His fusion protein was determined by immunohistochemistry with an anti-MYC antibody and the anti-CNGB3 antibody (clone 3B2) described above. To test binding of z13 peptide to CNGB3–MYC, FITC-z13 was added (5 μg ml^−1^) to the medium of HeLa cells transfected with the above-described vector and incubated at 37 °C for 15 min. Cells were washed with PBS, fixed with 4% PFA in PBS and observed under a fluorescence microscope.

### Binding kinetics of FITC-z13 to CNGB

A synthetic MYC epitope peptide (Anaspec) was biotinylated using sulpho-NHS-biotin (Pierce) and purified using a reverse-phase high-performance liquid chromatography column eluted with a linear gradient from 30 to 70% acetonitrile in water containing 0.1% trifluoroacetate over 20 min using Shimadzu SCL-10A HPLC. Biotin-MYC was bound to streptavidin-coated 96-well plates (Pierce), and MYC levels were measured by enzyme-linked immunosorbent assay (ELISA) using rabbit anti-MYC antibody and peroxidase-conjugated anti-rabbit antibody followed by a peroxidase colour reaction with TMB ELISA substrate solution (eBioscience). Levels of CNGB-MYC expressed by CNGB3–MYC-transfected HEK293T cells were measured by ELISA using biotin-MYC as the standard. Binding of FITC-z13 to CNGB3 was measured by adding serially diluted FITC-z13 to CNGB3-expressing HEK293T cells in 96-well plates and incubation at 37 °C for 60 min. Unbound FITC-z13 was washed with PBS and fluorescence remaining in the well was quantitated using a Beckman DTX880 plate reader. Binding kinetics was analysed by GraphPad Prism v6.0b software.

### Targeted apoptosis by dKLAK-z13 and HLAH-z13

Each peptide was dissolved in dimethylsulphoxide at 100 μg μl^−1^. Each peptide was added to the medium of cells, and the cells were cultured at 37 °C for 20 h. Apoptosis of CNGB3–MYC cDNA-transfected HEK293T cells was determined by TUNEL assay using the ApopTag immunoperoxidase kit (Chemicon, Temecula, CA).

### Targeted apoptosis of peritoneal endometriosis in the baboon

The protocol and use of the baboon model of endometriosis were approved by the IACUC of Texas Biomedical Research Institute. Three female baboons with advanced endometriosis were identified based on clinical symptoms, which include infertility and behaviour consistent with the inability to conceive, namely, dysmenorrhoea and extended menses. Candidate baboons also received an ultrasound as part of screening for study assessment. Under satisfactory anaesthesia, abdominal cavity was insufflated using a 1.5 l min^−1^ insufflator and Veress needle until the abdomen is distended and tympanic on percussion. A 5-mm diagnostic laparoscope was used to inspect pelvic organs. PBS (300 ml) was injected into the peritoneal cavity, adhesions were dissociated and the peritoneum washed. Three baboons received PBS containing a mixture of dKLAK-z13 (100 μg ml^−1^) and HLAH-z13 (25 μg ml^−1^) in 150 ml through a laparoscope into the peritoneum. Gas was evacuated from the peritoneum and the incisions sutured. Baboons were left ~20 h after administration and killed for tissue collection. Tissues were fixed with buffered formalin, and paraffin sections were made for histopathological analysis including TUNEL assay.

### Quantitative TUNEL analysis

Endometriosis lesions were identified in paraffin-embedded sections stained by hematoxylin and eosin. Endometrial glands were confirmed by immunostaining with an anti-keratan sulphate antibody (5D4)^40^ and an anti-estrogen receptor antibody^41^. The number of glands counted for each baboon ranged from 5 to 26. A gland was scored as TUNEL positive if it contained any TUNEL-positive cells. The number of TUNEL-positive glands in total glands was expressed as relative TUNEL positivity (%). Statistical analysis was performed using unpaired Student’s two-tailed *t*-test using GraphPad Prism v.6 software.

## Additional information

**How to cite this article:** Sugihara, K. *et al.* Development of pro-apoptotic peptides as potential therapy for peritoneal endometriosis. *Nat. Commun.* 5:4478 doi: 10.1038/ncomms5478 (2014).

## Supplementary Material

Supplementary Information Supplementary Figures 1-9 and Supplementary Table 1
