# Supplementary material for: Development of pro-apoptotic peptides as potential therapy for peritoneal endometriosis
Source: Nat Commun. 2014 Jul 22;5:4478. doi: 10.1038/ncomms5478 (PMC4109024; doi:10.1038/ncomms5478)
Supplement: Supplementary Information — Supplementary Figures 1-9 and Supplementary Table 1 [file ncomms5478-s1.pdf]

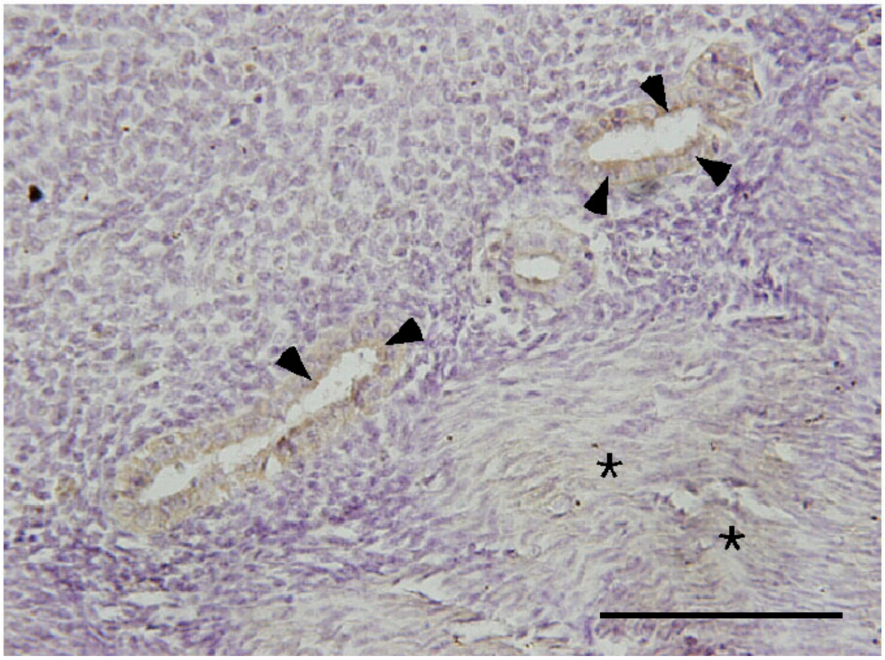

Supplemental Fig. 1. Phage overlay on frozen sections of human uterine endometriosis. Phage pools obtained after three rounds of screening were overlayed on frozen sections. Binding of phage to tissue was visualized by the immunoperoxidase method using rabbit anti-T7 phage antibody. Hematoxylin served as counterstain. Arrowheads show positive staining at apical membranes of endometrial glandular epithelia. Asterisks show weak staining of smooth muscle of the myometrium. Scale bar, 200 μm.

Trans-Proteomic Pipeline pepXML Viewer — Page 1 of 1

Restore Original | Trans-Proteomic Pipeline pepXML Viewer A.Keller 2.23.05 | Write Displayed Data Subset to File — file name: .shtml

Filter / Sort / Discard checked entries — More Analysis Info — Help

sort by: index spectrum peptide protein SEQUEST xcorr PeptideProphet™ probability

min probability: 0.8 min num tol term: exclude charge: 1+ 2+ 3+ others

SEQUEST results: min xcorr: min deltacn: max sprank:

proteins: ref all show all search scores: show hide Pep3D menu: show hide

include aa: mark aa: NxS/T: spectrum text: export to excel:

full menu show discarded entries clear manual discards/restores

3 entries retrieved from /home/sorcerer/output/2006-04-07 09h16m10s/interact.xml

index	prob	spectrum	xcorr	deltacn	sprank	m ions	peptide	protein
3	0.9995	sample539.27.27.2	1.494	0.200	4	7/ 16	R.SETDTDSEK.D	IPI:IPI00477539.1|ENSEMBL:ENSP0000035
19	0.9999	sample539.78.78.2	1.501	0.163	1	7/ 12	R.QRTALYK.K	IPI:IPI00178093.3|SWISS-PROT:Q9NQW8-1|REFSEQ_NP:NP_061971|ENSEMBL:ENSP000
51	1.0000	sample539.960.960.3	1.752	0.171	3	15/ 40	R.IFGEDFIATFK.R	IPI:IPI00307820.3|SWISS-PROT:Q96MM6|REFSEQ_NP:NP_443202|ENSEM INV:HIT000013326|VEGA:OTTHUMP0000003

Supplemental Fig. 2. Trans-Proteomic Pipeline pepXML view of top 3 proteins identified by SEQUEST Sorcerer 2 search engines. Three proteins were identified by proteomics analysis as potential receptors for z13 peptide. They included heat shock protein (70 kDa), CNGB3 (68 kDa), and NRDE2 homolog (133 kDa). We assumed that the receptor was a plasma membrane protein; thus we excluded both heat shock protein, which is cytoplasmic, and NRDE2, which is a nuclear protein, as candidates.

	A	B	C	D	E	F	G	H	I	J
1	spectrum	protein	protein_descr	peptide	pep_mass	MZratio	xcorr	deltacn	assumed_charge	ions
2	sample539.00078.00078.2	IPI00178093	Q9NQW8-1:Splice Isoform 1 of Cyclic nucleotide-gated cation channel beta 3	R.QRTALYK.K	878.4974	440.256	1.499	0.156	2	7/12

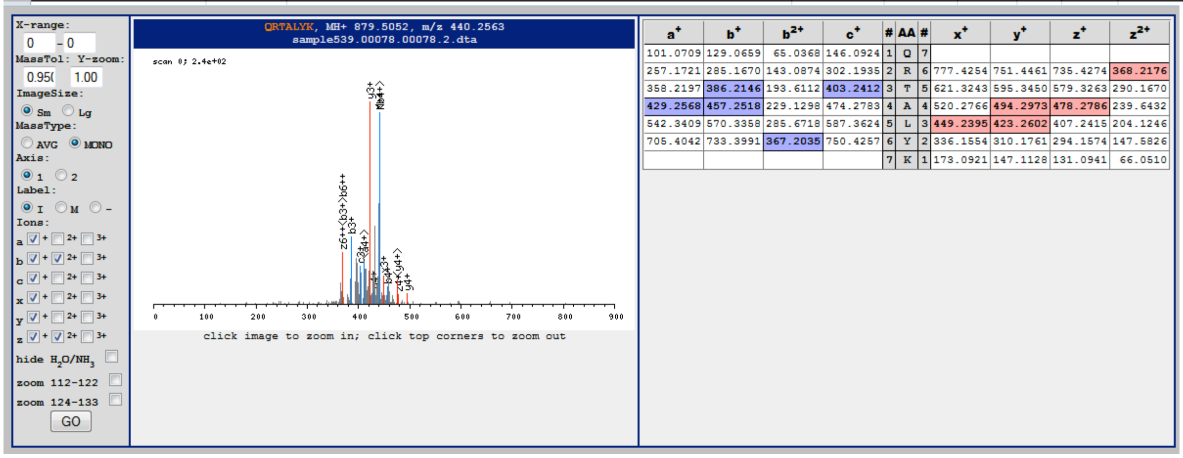

a^+	b^+	b^{2+}	c^+	#	AA	#	x^+	y^+	z^+	z^{2+}
101.0709	129.0659	65.0368	146.0924	1	Q	7				
257.1721	285.1670	143.0874	302.1935	2	R	6	777.4254	751.4461	735.4274	368.2176
358.2197	386.2146	193.6112	403.2412	3	T	5	621.3243	595.3450	579.3263	290.1670
429.2568	457.2518	229.1298	474.2783	4	A	4	520.2766	494.2973	478.2786	239.6432
542.3409	570.3358	285.6718	587.3624	5	L	3	449.2395	423.2602	407.2415	204.1246
705.4042	733.3991	367.2035	750.4257	6	Y	2	336.1554	310.1761	294.1574	147.5826
				7	K	1	173.0921	147.1128	131.0941	66.0510

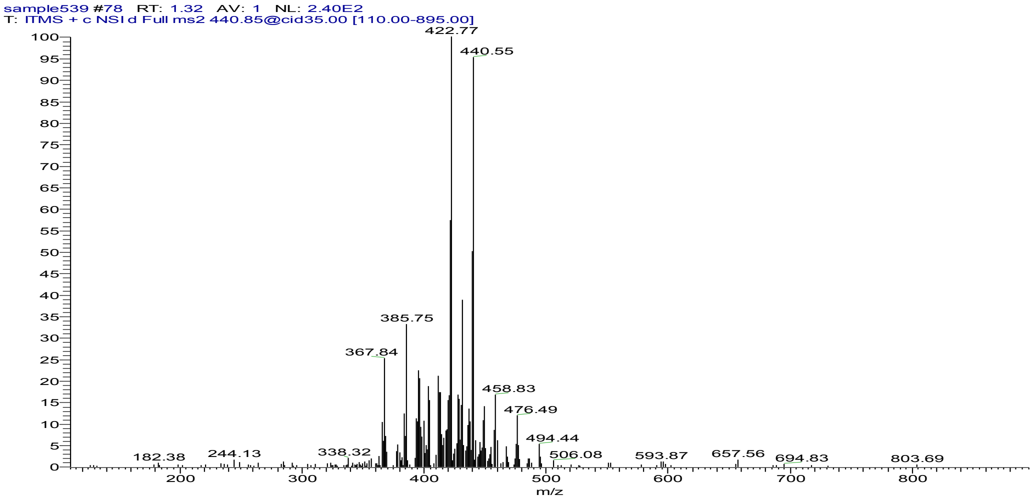

Supplemental Fig. 3. The MS2 spectra for the identified CNGB3 peptide. The MS2 spectra (upper panel) and distribution of the fragmentation ions for the identified peptide uniquely derived from CNGB3 (lower panel) are shown.

Human CNGB3 (809 aa)

MFKSLTKVNKVKPIGENNENEQSSRRNEEGSHPSNQSQQTTAQEENKGEEKSLKTKSTP
VTSEEPHTNIQDKLSKKNSSGDLTTNPDPQNAAEPTGTVPEQKEMDPGKEGPNSPQNKP
PAAPVINEYADAQLHNLVKRMRQRTALYKKKLVEGDLSSPEASPQTAKPTAVPPVKESD
DKPTEHYYRLLWFKVKKMPLTEYLKRIKLPNSIDSYTDRLYLLWLLLVTLAYNWNCWFI
PLRLVFPYQTADNIHYWLIADIICDIIYLYDMLFIQPRLQFVRGGDIIVDSNELRKHYR
TSTKFQLDVASIIPFDICYLFFGFNPMFRANRMLKYTSFFEFNHHLESIMDKAYIYRVI
RTTGYLLFILHINACVYYWASNYEGIGTTRWVYDGEGNEYLRCYYWAVRTLITIGGLPE
PQTLFEIVFQLLNFFSGVFVFSSLIGQMRDVIGAATANQNYFRACMDDTIAYMNNYSIP
KLVQKRVRTWYEYTWDSQRMLDESDLLKTLPTTVQLALAIDVNFSIISKVDLFKGCDTQ
MIYDMLLRLKSVLYLPGDFVCKKGEIGKEMYIIKHGEVQVLGGPDGTKVLVTLKAGSVF
GEISLLAAGGGNRRTANVVAHGFANLLTLDKKTLQEILVHYPDSERILMKKARVLLKQK
AKTAEATPPRKDLALLFPPKEETPKLFKTLLGGTGKASLARLLKLKREQAAQKKENSEG
GEEEGKENEDKQKENEDKQKENEDKGKENEDKDKGREPEEKPLDRPECTASPIAVEEEP
HSVRRTVLPRGTSRQSLIISMAPSAEGGEEVLTIEVKEKAKQ

Tryptic peptide sequence identified by proteomics.

3B2 epitope: a cytoplasmic domain peptide sequence used for raising mouse monoclonal antibody.

Supplemental Fig. 4. Peptide sequence identified by proteomics and 3B2 epitope for mouse CNGB3 monoclonal antibody.

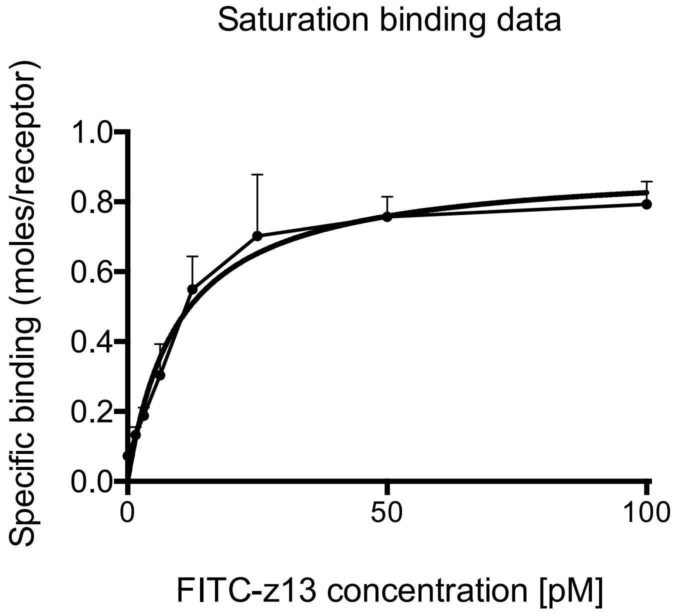

	Specific binding (moles/receptor)
Bmax	0.9068
Kd	9.759

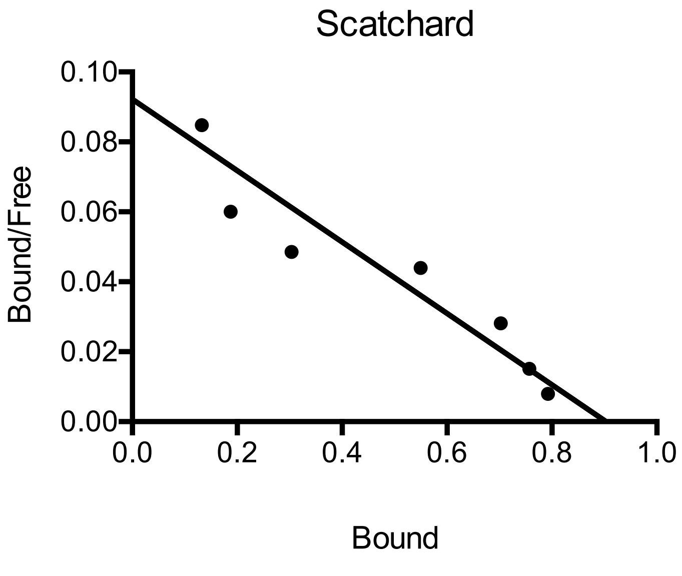

Supplemental Fig. 5. Kinetic analysis of FITC-z13 binding to HEK293T cells transfected for CNGB3-Myc.

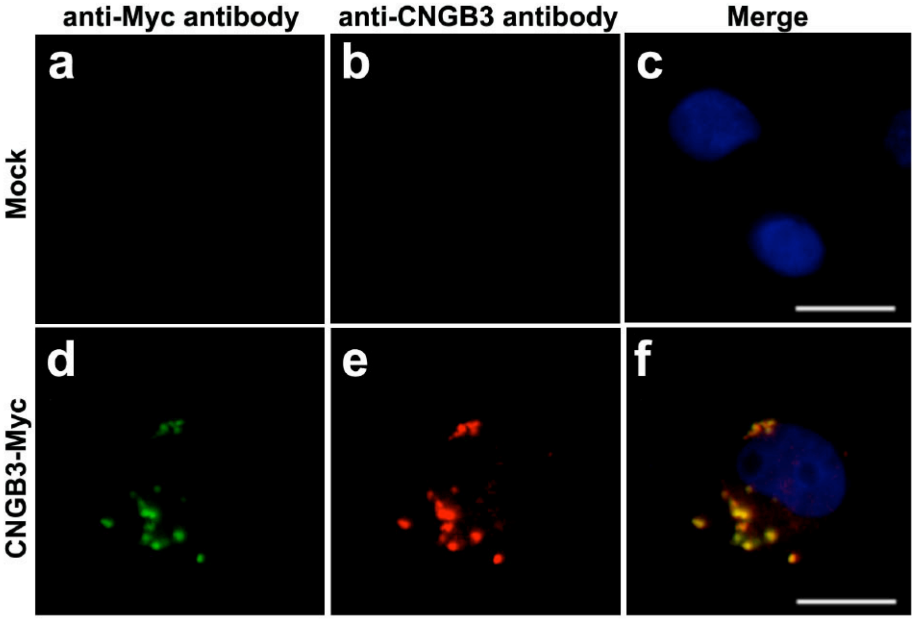

Supplemental Fig. 6. Immunostaining of transfected HeLa cells by anti-Myc antibody and anti-CNGB3 antibody. HeLa cells were transfected by either pCMV-Myc vector (mock) or with vector harboring CNGB3-Myc cDNA. Cells were subjected to double-immunofluorescence microcopy by rabbit anti-Myc antibody and mouse anti-CNGB3 antibody (3B2) followed by Alexa 488-conjugated anti-rabbit IgG antibody (green) and Alexa 594-conjugated anti-mouse IgG antibody (red). Nuclear staining were with DAPI (blue). Scale bar 50 μm.

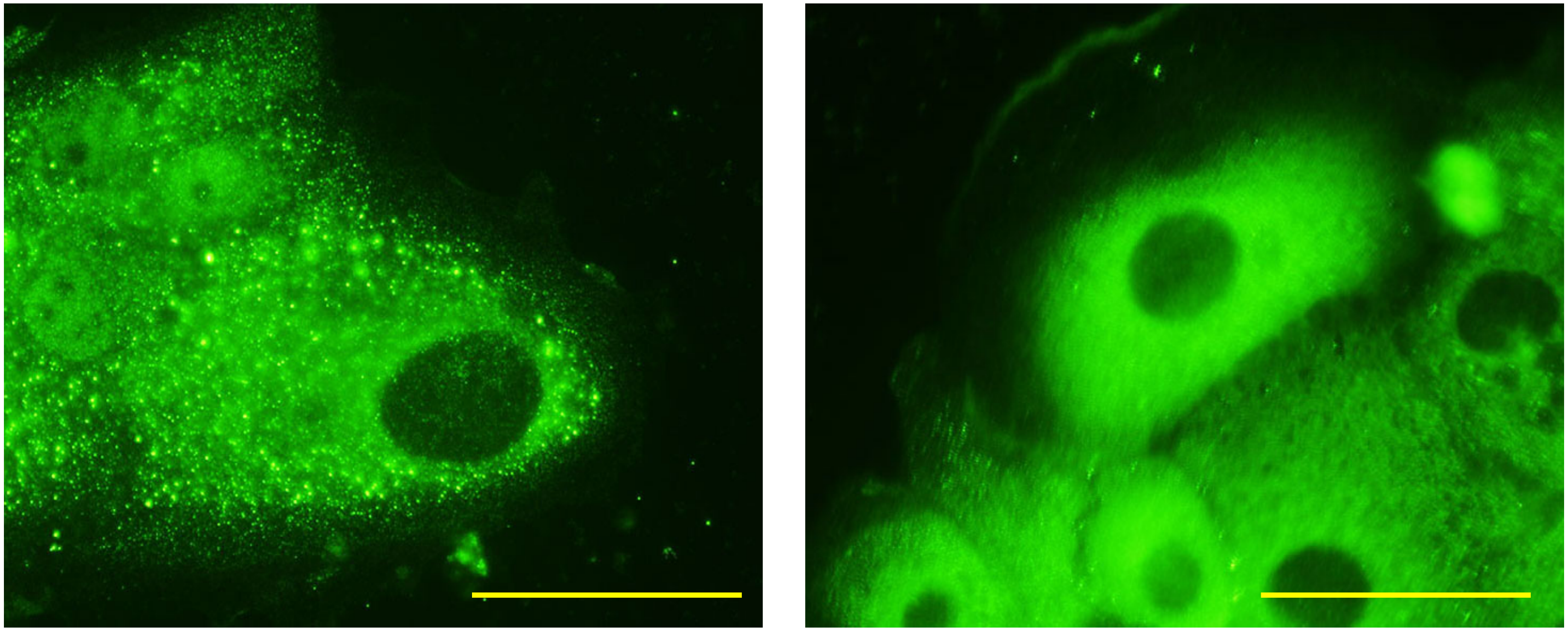

Supplemental Fig. 7. Localization of the known lysosomal marker protein ACP1 (acid phosphatase 1) in CNGB3 expressing A431 cells in the presence (right) or absence of HLA-z13, as an indicator of endosome-escaping activity. Cells on the right were treated with HLA-z13 (50 μg/ml) at 37 ^{o}C for 1 hour. Scale bars: 50 μm.

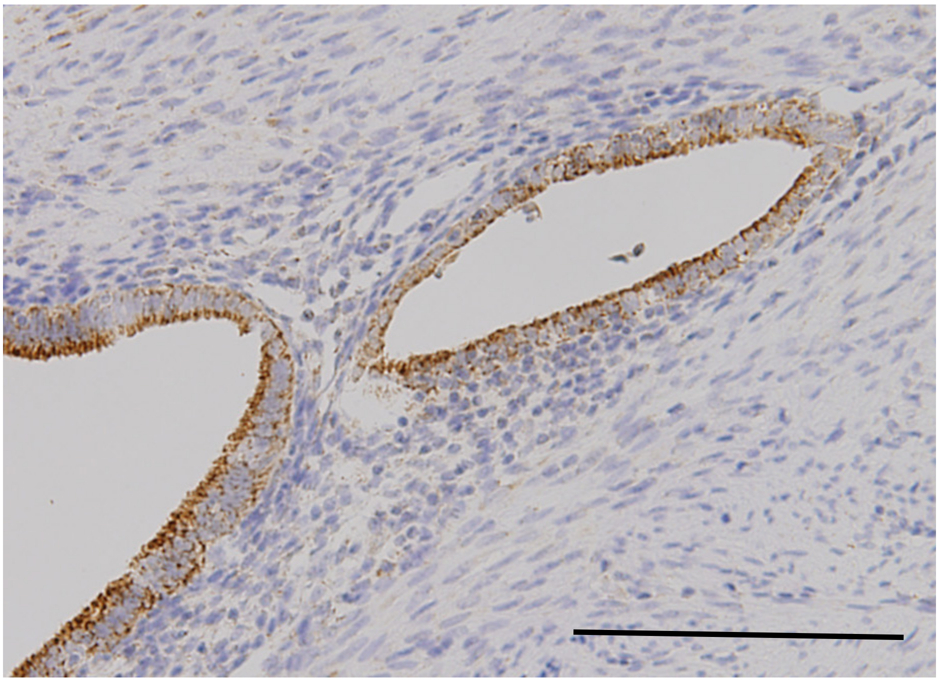

Supplemental Fig. 8. Immunohistochemistry of baboon endometriosis tissue stained by anti-CNGB3 antibody. Scale bar 100 μm

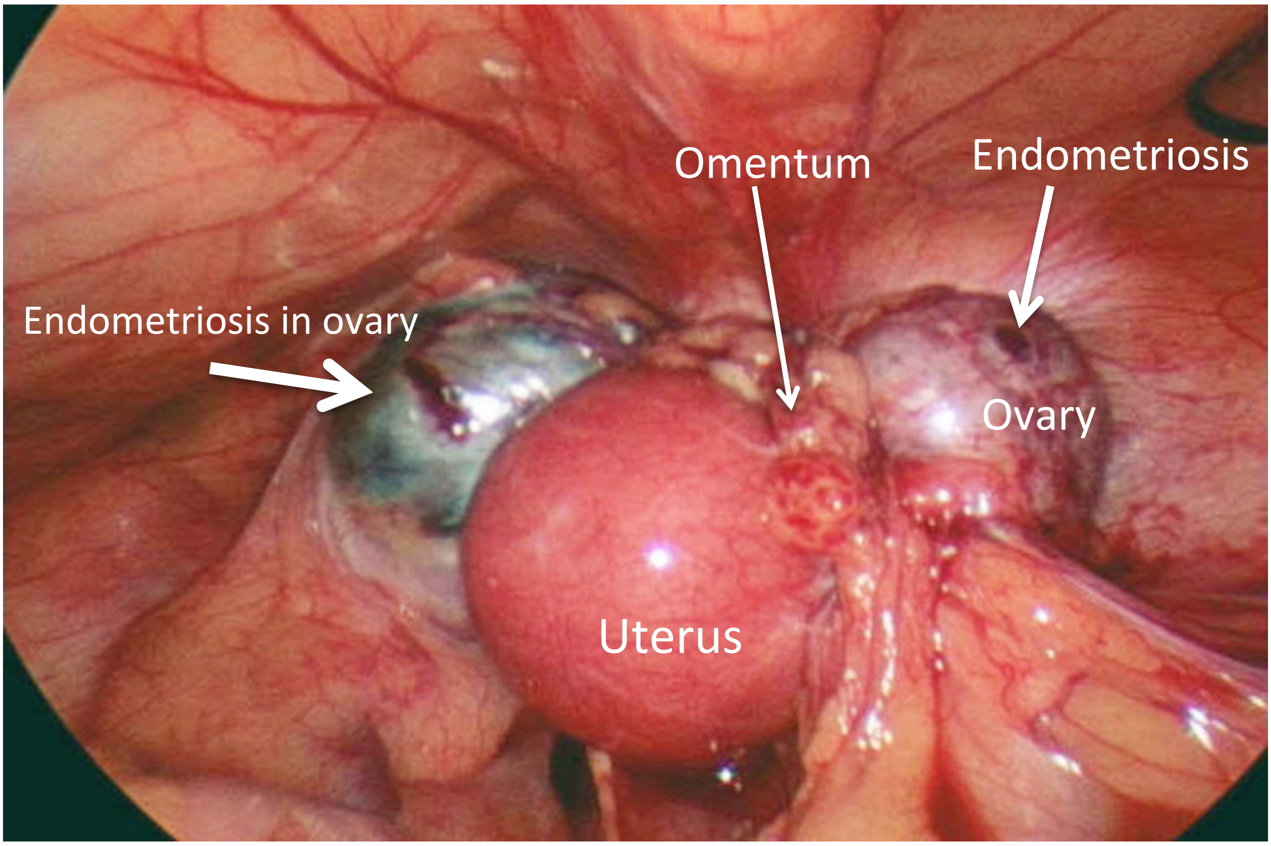

Supplemental Fig. 9. Peritoneal endometriosis in a baboon visualized by laparoscopy.

entry no.	protein	description	peptide sequence	Xcorr	deltacn	sprank	ions	protein probability
1	IPI00307820	Heat shock 70 kDa protein 12B.	IFGEDFIATFKR	1.752	0.171	3	15/ 40	1
2	IPI00178093	cyclic nucleotide-gated cation channel beta-3	QRTALYKK	1.501	0.163	1	7/ 12	0.9999
3	IPI00477539	Protein NRDE2 homolog	SETDTDSEK	1.494	0.2	4	7/16	0.9995

Supplemental Table 1. Identification of proteins by LC-MSMS analysis. Only one peptide sequence for each protein was available due to the small amount of protein isolated by peptide affinity chromatography. We validated that CNGB3 is the z13 receptor by biochemical assays shown in Figs. 2 and 3.
